# Concordance of Results from Randomized and Observational Analyses within the Same Study: A Re-Analysis of the Women’s Health Initiative Limited-Access Dataset

**DOI:** 10.1371/journal.pone.0139975

**Published:** 2015-10-06

**Authors:** Mark J. Bolland, Andrew Grey, Greg D. Gamble, Ian R. Reid

**Affiliations:** Department of Medicine, University of Auckland, Auckland, New Zealand; University of North Carolina School of Medicine, UNITED STATES

## Abstract

**Background:**

Observational studies (OS) and randomized controlled trials (RCTs) often report discordant results. In the Women’s Health Initiative Calcium and Vitamin D (WHI CaD) RCT, women were randomly assigned to CaD or placebo, but were permitted to use personal calcium and vitamin D supplements, creating a unique opportunity to compare results from randomized and observational analyses within the same study.

**Methods:**

WHI CaD was a 7-year RCT of 1g calcium/400IU vitamin D daily in 36,282 post-menopausal women. We assessed the effects of CaD on cardiovascular events, death, cancer and fracture in a randomized design- comparing CaD with placebo in 43% of women not using personal calcium or vitamin D supplements- and in a observational design- comparing women in the placebo group (44%) using personal calcium and vitamin D supplements with non-users. Incidence was assessed using Cox proportional hazards models, and results from the two study designs deemed concordant if the absolute difference in hazard ratios was ≤0.15. We also compared results from WHI CaD to those from the WHI Observational Study(WHI OS), which used similar methodology for analyses and recruited from the same population.

**Results:**

In WHI CaD, for myocardial infarction and stroke, results of unadjusted and 6/8 covariate-controlled observational analyses (age-adjusted, multivariate-adjusted, propensity-adjusted, propensity-matched) were not concordant with the randomized design results. For death, hip and total fracture, colorectal and total cancer, unadjusted and covariate-controlled observational results were concordant with randomized results. For breast cancer, unadjusted and age-adjusted observational results were concordant with randomized results, but only 1/3 other covariate-controlled observational results were concordant with randomized results. Multivariate-adjusted results from WHI OS were concordant with randomized WHI CaD results for only 4/8 endpoints.

**Conclusions:**

Results of randomized analyses in WHI CaD were concordant with observational analyses for 5/8 endpoints in WHI CaD and 4/8 endpoints in WHI OS.

## Introduction

The role that observational studies reporting effects of treatments should play in informing clinical practice is debated. Marked differences in the results of high-profile randomized controlled trials (RCTs) and observational studies have led to questions about the reliability of results of observational studies. The observational Nurses’ Health Study reported that use of oestrogen with or without progesterone was associated with a substantial reduction in the risk of cardiovascular disease in post-menopausal women [1, 2]. However in two large RCTs, women randomly allocated to oestrogen and progesterone treatment had increases in risk of cardiovascular disease [3, 4]. Similarly, observational studies suggested benefits for antioxidants on cancer prevention [5] and folic acid/ B vitamins for cardiovascular disease [6], but later RCTs reported either harms [7, 8] or no benefits [9–11] from these agents. In contrast, results from systematic reviews show generally good agreement between results from observational studies and those from RCTs [12–14]. However, within these systematic reviews, discrepancies did occur and substantial differences in the estimated magnitude of treatment effect between the different study designs were common [14]. For example, 62% of observation and randomized studies on the same topic had a >50% difference in the odds ratio [14].

There are many potential reasons for differences in results between observational studies and RCTs. They might result from differences in study design- for example, study populations may differ; RCTs are usually smaller and may not detect small effects; and RCTs usually involve shorter treatment exposure. Other differences might arise through confounding and bias in observational studies. Users of dietary supplements are generally healthier and of higher socioeconomic status than non-users, and these factors are often difficult to control for in statistical analyses. Thus, some of the benefits observed in the observational studies for such agents may reflect underlying health differences between people who use supplements and those who do not, even though attempts were made to adjust for such differences in statistical models.

The Women’s Health Initiative Calcium and Vitamin D trial (WHI CaD) represents a unique opportunity to explore differences in results between observational studies and RCTs. WHI CaD was a very large, long duration RCT that permitted the non-protocol use of study agents: women were randomly assigned to CaD or placebo, but were permitted to use personal calcium and vitamin D supplements. At randomization, 57% of participants were using either personal calcium or vitamin D supplements. Thus, it is possible to compare results from the two different study designs *within the same study*: a randomized design comparing the effects of CaD with placebo in women not using personal calcium or vitamin D supplements, and an observational design restricted to the placebo group comparing outcomes in women using personal calcium and vitamin D supplements with outcomes in non-users. Whether the results from these two different study designs are concordant or not might provide insights into differences between results from observational studies and RCTs.

## Methods

### WHI CaD trial

The design and results of the WHI CaD trial have been published in full [15–19]. The WHI clinical trials programme consisted of 3 trials. At entry to the programme, women were invited to take part in the WHI dietary modification trial, the WHI hormone therapy trial, or both. At their first or second annual follow-up visit, participants in these trials were invited to take part in WHI CaD. 36,282 post-menopausal women were randomized to daily supplemental calcium (1g) and vitamin D (400 IU) or matching placebos and followed for an average of 7y. Personal calcium supplements of up to 1g daily, and personal vitamin D supplements of up to 600 IU daily (and later 1000 IU daily) were permitted in WHI CaD [15]. Outcomes for cardiovascular events, hip and total fracture, colorectal, breast, endometrial and ovarian cancer, and mortality were adjudicated centrally, while other cancers were adjudicated by local researchers [20]. CaD had no effect on the incidence of hip or total fracture, cardiovascular outcomes, colorectal or breast cancer, or mortality [15–19]. We obtained the WHI limited-access clinical trials dataset from the National Heart Lung and Blood Institute (NHLBI). Data are anonymous in the dataset. A protocol was submitted to the NHLBI before any analyses were carried out. We attempted to replicate the approach of the WHI investigators where possible. Our re-analysis was approved by the Northern X regional ethics committee.

### Randomized study design analyses

We assessed the effects of CaD on myocardial infarction, stroke, all-cause mortality, hip and total fracture, and breast, colorectal, and total cancer (total cancer excludes non-melanoma skin cancer). Using an intention-to-treat approach, the effect of CaD on the time since randomization to the first event for each of these endpoints was assessed using Cox proportional hazards models, stratified by age, randomization status in the WHI hormone and dietary modification trials and relevant prevalent disease at baseline (history of breast, colorectal, or any cancer for breast, colorectal and total cancer endpoints respectively; and history of fracture for hip and total fracture; and history of cardiovascular disease for myocardial infarction and stroke). These analyses were performed in the cohort of participants who were not using personal non-protocol calcium or vitamin D supplements at randomization. We also performed these analyses in the entire WHI CaD cohort for comparison with the original publications.

### Observational study design analyses

We restricted analyses to the placebo group and compared outcomes in women using personal calcium and vitamin D supplements at randomization with women not using either personal calcium or vitamin D supplements at randomization for each of the above endpoints using Cox proportional hazards models as described for the randomized design. Because there were differences in baseline characteristics between supplement users and non-users, we carried out unadjusted and age-adjusted analyses, and analyses that controlled for other covariates. For multivariate analyses, we included variables that differed between the groups and/or might be potentially related to the outcome with the final model selection based on plausibility, parsimony, and consideration of similar models used by the WHI investigators [21]. We also used propensity scores to control for baseline differences. We used a stepwise logistic regression model that selected 52 of 478 baseline variables to create a propensity score for baseline personal use of calcium and vitamin D supplements that was included as a covariate in the Cox proportional hazards models. Finally, we performed analyses in which users of personal calcium and vitamin D supplements were matched with non-users based upon their propensity score. 5363 matched pairs were identified with propensity scores that differed by ≤0.07: the mean difference in propensity score for the pairs was 0.0041.

The WHI investigators reported analyses based on use of personal calcium and vitamin D supplements in the prospective WHI Observational Study (OS) which was recruited from the same catchment population as WHI CaD [21]. They compared outcomes over 7.2y for 15,476 women taking ≥500mg/d calcium and ≥400IU/d vitamin D at baseline with 23,561 women not using these supplements for cardiovascular, fracture, mortality and cancer endpoints [21]. We compared the results from our analyses with these previously published results.

### Concordance of results

There are no accepted criteria for defining concordance of results between studies. The point estimates of the hazard ratios for the treatment effects of CaD on the major outcomes in WHI CaD ranged from 0.88–1.08 with 95% confidence intervals spanning approximately ±0.15 [15–19]. We think a difference of 0.15 between hazard ratios is a reasonable threshold for concordance because smaller differences have little effect on absolute risk, and are therefore of less clinical relevance to individual patients. For these reasons, we considered results from the two study designs concordant when the absolute difference between the point estimates of the treatment effect is ≤0.15.

### Data and statistical analyses

We have reported the baseline characteristics at the time of randomization to CaD, whereas the WHI investigators reported these characteristics at the time of entry to the WHI programme. For body mass index, and dietary and supplemental calcium and vitamin D intakes, we used the latest value recorded between screening and one month following CaD randomization. Cox proportional hazards models and logistic regression were undertaken as described above using the SAS software package (SAS Institute, Cary, NC version 9.4). We matched personal users of calcium and vitamin D supplements by propensity score with the %gmatch macro in SAS [22]. The assumption of proportional hazards was explored by performing a test for proportionality of the interaction between variables included in the model and the logarithm of time. All tests were two-tailed and P<0.05 was considered significant.

## Results

At randomization, 43% of participants were not using personal calcium or vitamin D supplements, 54% were using personal calcium, 47% personal vitamin D, and 44% both personal calcium and vitamin D. For our analyses, the randomized design included the 15,646 (43%) participants not using personal calcium or vitamin D supplements. The observational design included the 15,828 (44%) participants from the placebo group who were either using both personal calcium and vitamin D or were not using either of these supplements at randomization. Baseline characteristics for the entire cohort and for the subgroups defined by treatment allocation and personal supplement use are shown in Table 1. The subgroups for the randomized design were well-matched for these baseline characteristics, whereas for the observational design, there were a number of important differences between the subgroups, including for variables such as age, body mass index, race, hormone replacement therapy use and history of medical conditions such as hypertension and fracture.

**Table 1:** Characteristics at randomization in the entire cohort, and in subgroups defined by treatment allocation and by use of personal calcium or vitamin D.

	Entirecohort	Randomized design^a^		Observational design^b^	
		CaD	Placebo	Personalcalcium andvitamin D	No personalcalcium orvitamin D
	n = 36282	n = 7891	n = 7755	n = 8073	n = 7755
**Age (y)**	63.5 (6.9)	62.8 (7.0)	62.9 (7.0)	64.0 (6.8)	62.9 (7.0)
**Body mass index (kg/m** ^**2**^ **)**	28.8 (5.8)	29.5 (5.9)	29.4 (6.0)	28.3 (5.7)	29.4 (6.0)
**Personal, non-protocol supplemental calcium intake (mg/d)**	314 (485)	0 (0)	0 (0)	554 (489)	0 (0)
**Dietary calcium intake (mg/d)**	815 (466)	801 (491)	790 (470)	831 (450)	790 (470)
**Personal, non-protocol supplemental vitamin D intake (μg/d)**	4.8 (5.9)	0 (0)	0 (0)	10.1 (4.4)	0 (0)
**Dietary vitamin D intake (μg/d)**	4.3 (3.1)	4.3 (3.2)	4.2 (3.2)	4.4 (3.0)	4.2 (3.2)
**Blood pressure (mmHg)**					
**Systolic**	126 (17)	126 (17)	126 (17)	125 (17)	126 (17)
**Diastolic**	74 (9)	75 (9)	75 (9)	74 (9)	75 (9)
**Medical history** ^c^					
**Current HRT use (trial/personal) (%)**	52	49	51	56	51
**High cholesterol requiring pills (%)**	12	12	12	12	12
**Cardiovascular disease (%)**	15	14	15	15	15
**Hypertension (%)**	33	33	35	49	35
**Stroke (%)**	1.0	1.0	1.2	1.0	1.2
**Myocardial infarction (%)**	1.8	2.2	2.0	1.5	2.0
**Any cancer (%)**	4	4	4	3.7	4.1
**Breast cancer (%)**	0.2	0.2	0.2	0.1	0.2
**Colorectal cancer (%)**	0.1	0.1	0.2	0.1	0.2
**Fracture ever (%)**	38	36	36	40	36
**Any fracture since 55y (%)**	15	14	14	16	14
**Hip fracture (%)**	2.4	2.0	1.9	3.0	1.9
**Diabetes (%)**	6	7	7	5	7
**Smoking status** ^c^					
**Never (%)**	52	52	52	53	52
**Past (%)**	40	39	38	41	38
**Current (%)**	8	9	9	6	9
**Race**					
**White**	83	78	78	88	78
**Black**	9	13	13	6	13
**Hispanic**	4	6	6	3	6
**Other**	3.4	3.4	2.9	3.5	2.9

Data are mean (SD) or %. HRT- hormone status. CaD- randomized to calcium plus vitamin D

^a^ Women not using personal calcium or vitamin D supplements at randomization

^b^ Women from the placebo group who were either using both personal calcium and vitamin D or were not using either of these supplements at randomization.

^c^ all data are at randomization except for medical history and smoking status which are at entry to Women’s Health Initiative clinical trials programme. 91% of participants in the calcium plus vitamin D trial entered the trial at their first annual visit in the clinical trials programme and the remainder at their second annual visit.

Personal supplement use tended to increase throughout the study. At their final study visit, 32% of participants in the entire cohort were not using personal calcium or vitamin D, and 60% were using both supplements. For the randomized design, 53% of participants in both groups continued to be non-users of personal calcium at their final visit. For the observational design, 14% of participants using personal calcium and vitamin D at randomization were no longer using these supplements at their final visit, and 53% of participants not using these supplements at randomization continued to be non-users at their final visit.

**Fig 1:**
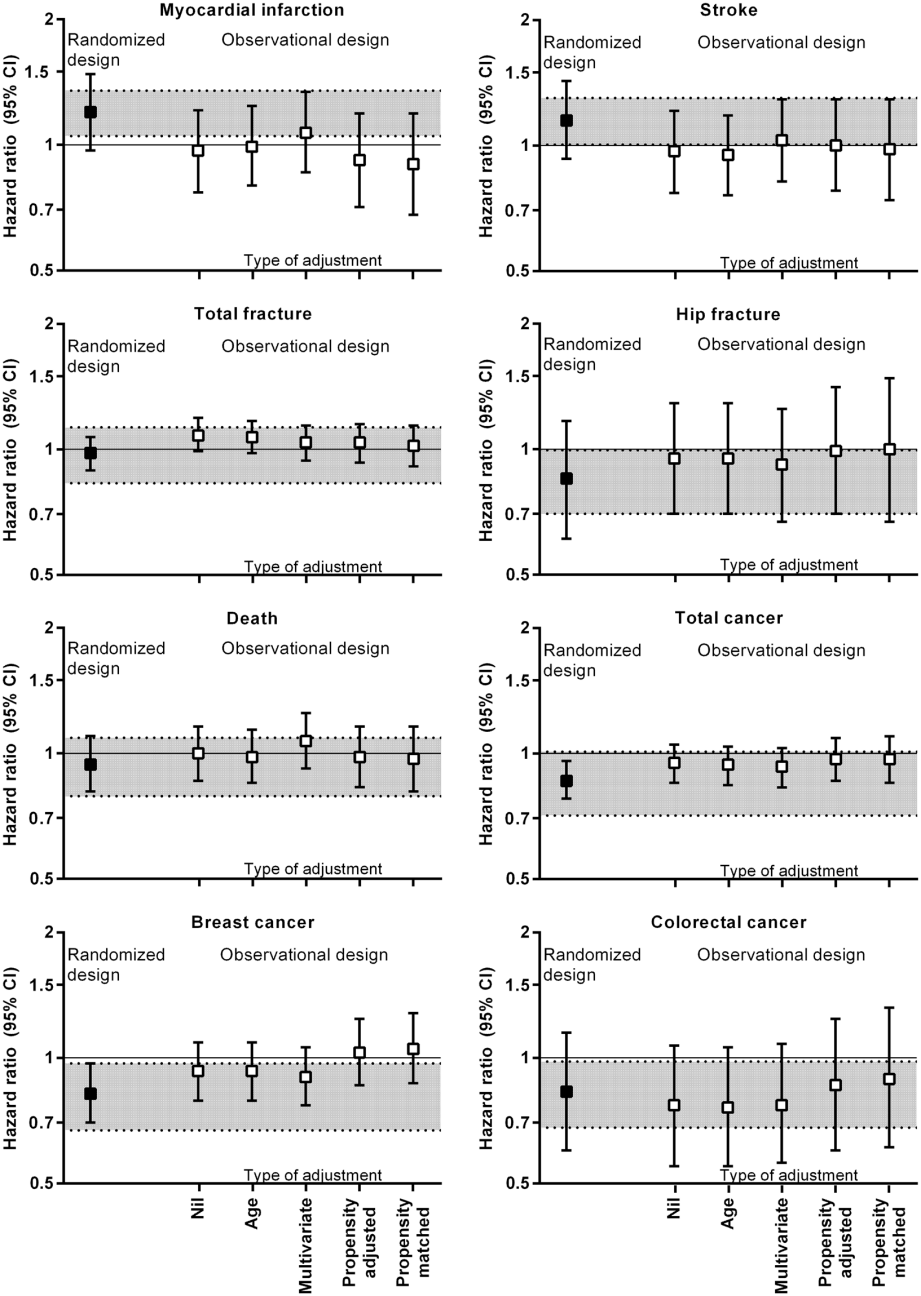
the effect of different methods of adjustment on results of observational analyses in comparison to the randomized design result. The dotted line indicates the concordance boundary (hazard ratio for randomized design ± 0.15).

Tables 2–4 and Fig 1 show the results for the randomized design, the observational design, and for comparison, the multivariate-adjusted results from the WHI OS. For myocardial infarction and stroke (Table 2), the results for the randomized and unadjusted observational designs were not concordant, and there was concordance with the randomized design results in only 2/8 analyses that controlled for covariates (age-, multivariate-adjusted, propensity-adjusted, or propensity-matched) observational analyses. The results of WHI OS were not concordant with the randomized design results.

**Table 2:** Comparison of results from the randomized and observational designs for cardiovascular events.

	Myocardial infarction		Stroke		Death	
**Entire cohort:**	**Comparison of randomization to CaD (N = 18,176) or placebo (18,106)**					
**Events (n)**	389/364		352/352		744/807	
**HR (95% CI)**	1.06 (0.92,1.23)		0.99 (0.85,1.15)		0.91 (0.83,1.01)	
**Randomized design:**	**Comparison of randomization to CaD (N = 7891) or placebo (7755) in women not using personal calcium or vitamin D**					
**Events (n)**	191/157		182/154		336/349	
**HR (95% CI)**	1.20 (0.97,1.48)		1.15 (0.93,1.43)		0.94 (0.81,1.10)	
**Observational design:**	**Comparison of users of personal calcium and vitamin D (N = 8073) with non-users (7755) in women allocated to placebo**					
**Events (n)**	162/157		157/154		360/349	
**HR (95% CI)**		**Agree** ^a^		**Agree** ^a^		**Agree** ^a^
**Unadjusted**	0.97 (0.77,1.21)	N	0.97 (0.77,1.21)	N	1.00 (0.86,1.16)	Y
**Age-adjusted**	0.99 (0.80,1.24)	N	0.95 (0.76,1.18)	N	0.98 (0.85,1.14)	Y
**Multivariate** ^b^	1.07 (0.86,1.34)	Y	1.03 (0.82,1.29)	Y	1.07 (0.92,1.25)	Y
**Propensity-adjusted** ^c^	0.92 (0.71,1.19)	N	1.00 (0.78,1.29)	N	0.98 (0.83,1.16)	Y
**Propensity-matched** ^d^	0.90 (0.68,1.19)	N	0.98 (0.74,1.29)	N	0.97 (0.81,1.16)	Y
**WHI OS results** ^e^	0.90 (0.75,1.09)	N	0.92 (0.77,1.09)	N	0.95 (0.85,1.06)	Y

Abbreviations: HR- hazard ratio. CI confidence interval.

^a^ Results from the observational design analyses are compared to the randomized design analysis, and are considered to agree when the difference between hazard ratios is ≤0.15.

^b^ adjusted for the variables in Table 5.

^c^ adjusted for a propensity variable derived from a stepwise logistic regression model that selected 52 of 478 baseline variables.

^d^ users of personal calcium and vitamin D supplements were matched to non-users based on the propensity variable

^e^ multivariable-adjusted results from the Women’s Health Initiative Observational Study (WHI OS) involving 46,892 women with average duration of follow-up 7.2y.

**Table 3:** Comparison of results from the randomized and observational designs for fracture.

	All fractures		Hip fracture	
**Entire cohort:**	**Comparison of randomization to CaD (N = 18,176) or placebo (18,106)**			
**Events (n)**	2102/2158		175/199	
**HR (95% CI)**	0.96 (0.91,1.02)		0.88 (0.72,1.08)	
**Randomized design:**	**Comparison of randomization to CaD (N = 7891) or placebo (7755) in women not using personal calcium or vitamin D**			
**Events (n)**	892/892		68/82	
**HR (95% CI)**	0.98 (0.89,1.07)		0.85 (0.61,1.17)	
**Observational design:**	**Comparison of users of personal calcium and vitamin D (N = 8073) with non-users (7755) in women allocated to placebo**			
**Events (n)**	1005/892		88/82	
**HR (95% CI)**		**Agree** ^a^		**Agree** ^a^
**Unadjusted**	1.08 (0.99,1.19)	Y	0.95 (0.70,1.29)	Y
**Age-adjusted**	1.07 (0.98,1.17)	Y	0.95 (0.70,1.29)	Y
**Multivariate** ^b^	1.04 (0.94,1.14)	Y	0.92 (0.67,1.25)	Y
**Propensity-adjusted** ^c^	1.04 (0.93,1.15)	Y	0.99 (0.70,1.41)	Y
**Propensity-matched** ^d^	1.02 (0.91, 1.14)	Y	1.00 (0.67, 1.48)	N
**WHI OS** ^e^	1.07 (1.01,1.14)	Y	0.88 (0.70,1.11)	Y

^a^ Results from the observational design analyses are compared to the randomized design analysis, and are considered to agree when the difference between hazard ratios is ≤0.15.

^b^ adjusted for the variables in Table 5.

^c^ adjusted for a propensity variable derived from a stepwise logistic regression model that selected 52 of 478 baseline variables

^d^ users of personal calcium and vitamin D supplements were matched to non-users based on the propensity variable

^e^ multivariable-adjusted results from the Women’s Health Initiative Observational Study (WHI OS) involving 46,892 women with average duration of follow-up 7.2y.

**Table 4:** Comparison of results from the randomized and observational designs for cancer.

	Breast cancer		Colorectal cancer		Any cancer	
**Entire cohort:**	**Comparison of randomization to CaD (N = 18,176) or placebo (18,106)**					
**Events (n)**	673/691		169/162		18176/18106	
**HR (95% CI)**	0.97 (0.87,1.08)		1.04 (0.84,1.29)		0.97 (0.91,1.04)	
**Randomized design:**	**Comparison of randomization to CaD (N = 7891) or placebo (7755) in women not using personal calcium or vitamin D**					
**Events (n)**	261/310		67/82		633/715	
**HR (95% CI)**	0.82 (0.70,0.97)		0.83 (0.60,1.15)		0.86 (0.78,0.96)	
**Observational design:**	**Comparison of users of personal calcium and vitamin D (N = 8073) with non-users (7755) in women allocated to placebo**					
**Events (n)**	301/310		65/82		703/715	
**HR (95% CI)**		**Agree** ^a^		**Agree** ^a^		**Agree** ^a^
**Unadjusted**	0.93 (0.79,1.09)	Y	0.77 (0.55,1.07)	Y	0.95 (0.85,1.05)	Y
**Age-adjusted**	0.93 (0.79,1.09)	Y	0.76 (0.55,1.06)	Y	0.94 (0.84,1.04)	Y
**Multivariate** ^b^	0.90 (0.77,1.06)	Y	0.77 (0.56,1.08)	Y	0.93 (0.83,1.03)	Y
**Propensity-adjusted** ^c^	1.03 (0.86,1.24)	N	0.86 (0.60,1.24)	Y	0.97 (0.86,1.09)	Y
**Propensity-matched** ^d^	1.05 (0.87, 1.28)	N	0.89 (0.61, 1.32)	Y	0.97 (0.85, 1.10)	Y
**WHI OS** ^e^	1.12 (0.99,1.28)	N	0.83 (0.61,1,12)	Y	1.03 (0.95,1.11)	N

^a^ Results from the observational design analyses are compared to the randomized design analysis, and are considered to agree when the difference between hazard ratios is ≤0.15.

^b^ adjusted for the variables in Table 5.

^c^ adjusted for a propensity variable derived from a stepwise logistic regression model that selected 52 of 478 baseline variables.

^d^ users of personal calcium and vitamin D supplements were matched to non-users based on the propensity variable

^e^ multivariable-adjusted results from the Women’s Health Initiative Observational Study (WHI OS) involving 46,892 women with average duration of follow-up 7.2y.

**Table 5:** Variables included in multivariate analyses.

Variable	MyocardialInfarction/Stroke	Death	Any/Hipfracture	BreastCancer	ColorectalCancer	AnyCancer
Age	X	X	X	X	X	X
Body mass index	X	X	X	X	X	X
Systolic blood pressure	X					
Number of falls			X			
Hormone therapy use	X	X	X	X	X	X
Smoking	X	X	X	X	X	X
Alcohol intake	X	X	X	X	X	X
Race	X	X	X	X	X	X
Education level	X	X	X	X	X	X
Family Income	X	X	X	X	X	X
Region USA	X	X	X	X	X	X
Baseline history of						
Cardiovascular disease,myocardial infarctionor stroke	X	X				
Hypertension	X	X				
High cholesterol	X	X				
Diabetes	X	X				
Fracture		X	X			
Any cancer		X				X
Breast cancer				X		
Colon cancer					X	

Categorical variables: number of falls = 0, 1, 2, or ≥3 falls in past 12 months; hormone therapy use = current personal use or randomization to active treatment in WHI hormone therapy trial, or non-use; smoking = never, past, or current smoker; alcohol intake = non or past drinker, <1 drink/week, 1 to <7 drinks/week, ≥7 drinks/week; race- white, other; education level- beyond high school, other; family income- ≥$35,000/year, <$35,000/year; region USA- northeast, south, midwest, west.

In contrast, for death (Table 2), all of the unadjusted and covariate-controlled observational design results and the WHI OS result were concordant with the randomized design result. Similarly, for hip and total fracture (Table 3), the unadjusted observational design result, 7/8 of the covariate-controlled observational results, and the WHI OS result were concordant with the randomized design result.

For breast cancer (Table 4), the unadjusted, age- and multivariate-adjusted observational design results were concordant with the randomized design result. However, neither the WHI OS result nor the propensity-adjusted or propensity-matched observational design results were concordant with the randomized design result. For colorectal and any cancer (Table 4), the unadjusted and covariate-controlled observational design results were concordant with the randomized design results. However, only the WHI OS result for colorectal cancer was concordant with the randomized result.

In sensitivity analyses, we explored the effect of selecting different thresholds for defining concordance. If we adopted a threshold of ±0.10 for concordance, 3/8 unadjusted and 15/32 covariate-controlled observational design results, and 4/8 WHI OS results were concordant with the randomized design results. Using a threshold of ±0.20, 7/8 unadjusted and 26/32 covariate-controlled observational design results, and 5/8 WHI OS results were concordant with the randomized design results. (For the primary analyses with a threshold of ±0.15, the frequency of concordance was 6/8, 23/32, and 4/8, respectively).

## Discussion

There were different patterns of results from randomized and observational study designs for different outcomes in WHI CaD. For death, colorectal and total cancer, and hip and total fracture, results of unadjusted observational analyses were concordant with randomized design results, and adjustment for other variables in the observational analyses generally had little effect. For myocardial infarction and stroke, results of unadjusted observational analyses were not concordant with the randomized design results, and adjustment for other variables generally did not substantially decrease the differences between the results. For breast cancer, the unadjusted, age- and multivariate-adjusted observational results were concordant with the randomized results, but propensity adjustment or matching increased the differences between the results. Overall, 6/8 unadjusted, 6/8 age-adjusted, 8/8 multivariate-adjusted, 5/8 propensity-adjusted, and 4/8 propensity-matched observational results were concordant with the randomized results. In comparison, 4/8 results from the WHI OS were concordant with the randomized results.

The results suggest that *within the same study* there are not substantial differences between results from randomized and observational study designs. Other than for myocardial infarction and stroke, all the unadjusted observational results were concordant with the randomized design results, and results from all multivariate-adjusted results using Cox proportional hazard models incorporating potential confounders were concordant. Results from propensity-adjusted and propensity-matched models were generally similar to the multivariate Cox proportional hazard model results. However, there were small differences between these models for some endpoints (myocardial infarction and breast cancer), and the propensity-adjusted and propensity-matched models did not fall within the defined range for concordance for these two outcomes or for stroke. An important limitation is that the randomized and observational study designs were not independent because the control group was the same for both designs. This feature may have contributed to the smaller differences between the within-study observational and randomized design comparisons compared to the between-study comparisons.

Although there was fairly high concordance of observational and randomized design results within WHI CaD, concordance between the WHI CaD randomized results and the WHI OS results was only 50%, even though the two studies used similar methodology and recruited participants from the same population. Thus, differences in results between RCTs and observational studies may be due to differences between studies, even when they are small and subtle, rather than due to the specific design of the study (observational versus RCT). One potential difference is the willingness of participants to take part in a clinical trial and be randomized and blinded to a treatment. It is possible that responses to a treatment might be different in people willing to participate in a clinical trial compared to people unwilling to participate.

The results suggest that the influence of potential confounders may vary for different outcome variables and in different statistical models, although any such differences were small. There were substantial differences between users of personal calcium and vitamin D and those not taking either of these supplements for variables such as age, body mass index, and race which are all associated with cardiovascular disease, fractures, and cancer. Age and race were statistically significant predictors of fracture and cancer outcomes in our analyses, but adjustment for these and other variables did not have a substantial impact in any of the observational analyses, with all differences between the unadjusted and covariate-controlled effect estimates being <0.12. There were small differences between effect estimates from Cox proportional hazards models and propensity score-based models, but all differences were <0.17. When effect sizes are large, such differences are likely to have little impact. However, 70% of numeric associations were weak (odds ratio or relative risk between 0.5 and 2.0) in a recent survey of >2000 outcomes assessed in the influential observational Nurses’ Health Study [23]. For effect estimates of this magnitude, small effects from adjusting for potential confounders could have substantial impact. It is not certain what accounts for the different impacts of confounders on outcome variables, but it highlights the difficulties in carrying out and interpreting multivariate analyses. It suggests that multivariate analyses of observational studies should be treated as exploratory, with a number of different models and techniques applied. The results should be reported accordingly, rather than simply presenting the results from a single “best” model, as commonly occurs.

An important limitation of our analyses is that the effects of CaD on all the outcomes we measured in both the randomized and observational designs were weak, with all effect estimates ranging between 0.76 and 1.20. Although WHI CaD was a large study and the confidence intervals around the effect estimates were generally narrow, it is possible that results might differ for agents with stronger therapeutic effects. We are not aware of any other completed large studies with a similar study design- that is, the study permitted non-protocol use of the study medication and had a large proportion of non-protocol users at baseline. However, a large study of vitamin D supplements currently underway also permits the use of non-protocol vitamin D supplements [24]. This study may therefore allow a similar analysis to ours to be undertaken once the study is completed. Cross-over between the study groups occurred with non-users of supplements at baseline starting them during follow-up and also, less commonly, baseline users discontinuing supplements. This cross-over between groups may have obscured true effects of CaD. Finally, an important limitation is that our definition of congruence between study results is necessarily arbitrary, being based on clinical pragmatism [23], although we did explore other definitions in sensitivity analyses.

In summary, these results do not suggest that there are substantial differences between the results of randomized and observational study designs *within the same study*, although concordance of results did vary between outcomes. The comparison of randomized results from WHI CaD with those from the separate WHI OS observational study again highlight the inconsistency of results between RCTs and observational studies, even, in this case, when the studies used similar methodology in the analyses and recruited participants from the same population. The effect of adjusting for potential confounders in observational analyses differed by only small amounts in a range of outcome variables and in the different methods of adjustment used. However, as the effect estimates were also small, some of these differences did alter the conclusions as to whether results were concordant or not. This suggests that multivariate adjustment in observational studies should explore a variety of different models and techniques, and report the impact of the different approaches as exploratory analyses.
